# Chick Chorioallantoic Membrane as a Patient-Derived Xenograft Model for Uveal Melanoma: Imaging Modalities for Growth and Vascular Evaluation

**DOI:** 10.3390/cancers15051436

**Published:** 2023-02-24

**Authors:** Theodora Tsimpaki, Nikolaos E. Bechrakis, Berthold Seitz, Miriam M. Kraemer, Hongtao Liu, Sami Dalbah, Ekaterina Sokolenko, Utta Berchner-Pfannschmidt, Miltiadis Fiorentzis

**Affiliations:** 1 Department of Ophthalmology, University Hospital Essen, University of Duisburg-Essen, Hufeland Str. 55, 45147 Essen, Germany; 2 Department of Ophthalmology, Saarland University Medical Center, Kirrberger Str. 100, 66421 Homburg, Germany

**Keywords:** uveal melanoma, patient-derived xenograft, CAM assay, in vivo human uveal melanoma model, real-time imaging, OCT, angiography, ultrasound, image analysis

## Abstract

**Simple Summary:**

The objective of the present study is to establish a new in vivo patient-derived xenograft (PDX) model for uveal melanoma (UM) based on the chick chorioallantoic membrane (CAM) assay. Furthermore, various implantation techniques and monitoring tools, such as different ultrasound modalities, optical coherence tomography and angiography, fluorescein angiography, image analysis, as well as fluorescent immunohistochemistry, were investigated for the assessment of the growth, vascularity, and, therefore, the viability of the implanted grafts. The results suggested an increase in the size parameters of the UM specimens and comparable data for the applied monitoring instruments, resulting in the CAM-based PDX model becoming an alternative step in translational cancer research for novel diagnostic and therapeutic experimentation in the field of ocular melanoma.

**Abstract:**

Background: Patient-derived tumor xenografts (PDXs) have emerged as valuable preclinical in vivo models in oncology as they largely retain the polygenomic architecture of the human tumors from which they originate. Although animal models are accompanied by cost and time constraints and a low engraftment rate, PDXs have primarily been established in immunodeficient rodent models for the in vivo assessment of tumor characteristics and of novel therapeutic cancer targets. The chick chorioallantoic membrane (CAM) assay represents an attractive alternative in vivo model that has long been used in the research of tumor biology and angiogenesis, and can overcome some of these limitations. Methods: In this study, we reviewed different technical approaches for the establishment and monitoring of a CAM-based uveal melanoma PDX model. Forty-six fresh tumor grafts were acquired after enucleation from six uveal melanoma patients and were implanted onto the CAM on ED7 with Matrigel and a ring (group 1), with Matrigel (group 2), or natively without Matrigel or a ring (group 3). Real-time imaging techniques, such as various ultrasound modalities, optical coherence tomography, infrared imaging, and imaging analyses with Image J for tumor growth and extension, as well as color doppler, optical coherence angiography, and fluorescein angiography for angiogenesis, were performed on ED18 as alternative monitoring instruments. The tumor samples were excised on ED18 for histological assessment. Results: There were no significant differences between the three tested experimental groups regarding the length and width of the grafts during the development period. A statistically significant increase in volume (*p* = 0.0007) and weight (*p* = 0.0216) between ED7 and ED18 was only documented for tumor specimens of group 2. A significant correlation of the results for the cross-sectional area, largest basal diameter, and volume was documented between the different imaging and measurement techniques and the excised grafts. The formation of a vascular star around the tumor and of a vascular ring on the base of the tumor was observed for the majority of the viable developing grafts as a sign of successful engraftment. Conclusion: The establishment of a CAM-PDX uveal melanoma model could elucidate the biological growth patterns and the efficacy of new therapeutic options in vivo. The methodological novelty of this study, investigating different implanting techniques and exploiting advances in real-time imaging with multiple modalities, allows precise, quantitative assessment in the field of tumor experimentation, underlying the feasibility of CAM as an in vivo PDX model.

## 1. Introduction

Uveal melanoma (UM), as the most common primary intraocular tumor, represents 79–81% of all ocular melanomas, with an incidence of 2–8 new cases in Europe and 5 in the United States per million population per year [1,2]. It predominantly involves the choroid, as 83% of cases arise from the uvea and 5–10% from the conjunctiva and other sites [3]. It shows a wide variation in incidence depending on age, ethnicity, and geographic gradient [1,2,4]. An inverse association with the intensity of ultraviolet radiation has been observed, as the incidence of uveal melanoma increases from southern to northern latitudes in America, as well as in Europe [1,2,3]. The disease shows no gender preference, but there is a prevalence among older age groups, with a peak at approximately 70 years old [5,6]. The presence of fair complexion, oculodermal melanocytosis, familial BAP1 mutations, neurofibromatosis, dysplastic nevus syndrome, and iris or choroidal nevus are considered additional prognostic factors for the potential development of uveal melanoma [7,8].

In approximately 50% of UM cases, a complete chromosome 3 monosomy can be observed [9,10]. The five-year survival rate decreases from 90% to 39% for patients with monosomy 3 [11]. A mutation of the BRCA1-associated protein-1 (BAP1), as a tumor-suppressor gene placed on chromosome 3, has been detected in 47% of patients with primary UM [11,12]. Aberrations of chromosome 8, such as 8q gain, including trisomy 8, isochromosome 8q, and amplification of the c-myc gene, occur in approximately 40% of cases of UM, whereas 8p loss is rare [13,14]. Besides 3 loss and 8q gain, the detection of chromosome 1p loss and 6q loss also appears to be associated with poor prognosis [15]. Another genetic risk factor is mutations in G protein α subunits, specifically subunit αq (GNAQ) and G protein subunit α11 (GNA11) [16].

Prognosis is determined by various factors, including the tumor site and size, the age of the patient at the time of diagnosis, and the extent of metastatic disease, but especially the histological characteristics and molecular pathology of UM. The therapeutic plan comprises brachytherapy, teletherapy, and surgical excisions, such as transscleral resection, endoresection, and enucleation. In spite of the high disease control rate for primary tumors, a standardized treatment protocol for the selection of the optimal therapeutic options based on the specific tumor characteristics and a common consensus on surveillance for early detection are necessary. Despite advances in local tumor control, 52% of UM patients develop clinical metastases, and the 10-year mortality is about 43% [17,18]. Due to the rarity of UM and its high accompanying metastatic and mortality rate, reliable in vivo models are required for the investigation of the tumor’s behavior, its metastatic setting, and the efficacy of novel therapeutic targets.

Patient-derived experimental models include in vitro 2D cell cultures, 3D organoids, as well as in vivo xenografts in animals [19]. They largely recapitulate the inter- and intratumoral heterogeneity, showing an advantage over traditional models [20]. Patient-derived xenograft (PDX) models retain the histological and genetic characteristics of their donor tumor, resulting in them being an effective experimental platform for the characterization of pathophysiology and molecular features, as well as for the investigation of drug responsiveness [21,22]. In view of precision medicine, the preselection of responsive patients to therapeutic regimens is necessary to reduce morbidity and mortality [23]. PDX models offer the possibility of investigating such individualized treatments due to the accurate reflection of the complexity of the tumor microenvironment [24]. Furthermore, they are particularly crucial in the assessment of cancer types, with high variations in molecular alterations or in rare cancer entities, such as uveal melanoma. Immunodeficient rodents have most commonly been used for the establishment of in vivo PDX models [25]. Practical and scientific limitations are associated with the use of such murine preclinical models. Immense resources and prohibitively high costs are imperative to maintain a colony in an animal facility. Moreover, the extended time, the low engraftment rate for certain cancer types, as well as the ethical question underline the necessity for alternative in vivo experimentation models to overcome such shortcomings [19,22,26,27,28,29].

The chick chorioallantoic membrane (CAM) assay has emerged as a suitable and reproducible in vivo model in preclinical cancer research. The CAM is a highly vascularized extraembryonic membrane that is formed by the partial fusing of the chick’s chorion and its allantois during embryonal development and is connected to the embryo through a continuous circulatory system [22,30,31]. The embryo is not fully immunocompetent until day 18, which results in it being ideal for tissue grafting during the early development stages [19,32]. The CAM is a low-cost and easily accessible model that facilitates visualization and intervention during experimentation, although a non-specific inflammatory response could appear after day 15 [19,33,34]. The growth of cancer cells has been well-established in this in vivo model, which displays rapid engraftment with vascularization by chick vessels within 2–5 days after inoculation in comparison with other mammalian models [33]. Techniques that have been used to visualize and assess tumor growth in the CAM assay include the detection of human urokinase plasminogen activator, PCR amplification of human sequences, immunohistochemistry, ultrasound, MRI, PET/CT imaging, radiotracers, and viral nanoparticles [22,32,35,36,37]. There is a limited number of references in the literature regarding the use of CAM for the research of UM models [38,39,40]. Increasing interest has been shown in the CAM as a model for the research of not only neoplastic growth, but also the transplantation and maintenance of foreign tissue, enabling the establishment of patient-derived CAM xenografts for various cancer entities [22].

To our knowledge, the implantation of fresh patient-derived uveal melanoma xenografts onto the CAM for the establishment of a new PDX model has not yet been established. In this study, various implantation conditions were analyzed to optimize the engraftment of fresh patient-derived uveal melanoma specimens into the CAM and to evaluate their growth potential. Furthermore, multiple alternative monitoring techniques were applied and compared for the assessment of growth, extension, invasion, vascularization, and, therefore, viability. The results allow us to propose the CAM-based PDX model as a new tool to evaluate the properties of uveal melanoma in vivo, further facilitating its application in an individualized therapeutic context in the future.

## 2. Materials and Methods

### 2.1. Chicken Chorioallantoic Membrane Assay

Briefly, fertilized white Lohmann chicken eggs were cleaned with 50% ethanol to avoid potential contamination. The eggs were placed in an upright position in an incubator at a temperature of 37.5 °C and a humidity of approximately 60–70% to induce embryogenesis (Bruja 3000 digital, Siepmann, Germany and Mini Pro 147, Maino, Italy). On day five of embryonic development, the CAM was lowered by removing 4–6 or 8–10 mL albumin with a 10 mL sterile syringe and an 20-gauge safety butterfly cannula (Safety-Multifly Needle, Sarstedt, Nümbrecht, Germany). The puncture entrance was resealed with surgical tape (3M Micropore surgical tape, Saint Paul, MN, USA). On experimental day 6 (ED6), a window was cut in the shell of the eggs under aseptic conditions, exposing the embryonic structures. The aperture of the eggshell was subsequently covered with Parafilm (Bemis Company Inc., Neenah, WI, USA).

### 2.2. Patient-Derived Xenografts

Tumor specimens were harvested in the operating room from six advanced-stage uveal melanoma patients. The histopathological and genetic examination revealed a spindle cell uveal melanoma with disomy 3 in all patients. Each tumor sample was divided into 5–15 homogenous fragments of approximately 4 × 4 mm, depending on the tumor’s size, by the surgeon. These were immediately placed in 2 mL Eppendorf tubes with 500 µL RPMI (Gibco RPMI 1640 Medium, Thermo Fisher Scientific, Waltham, MA, USA) and preserved in ice until engraftment. The size dimensions, as well as the weight of the samples, were then accurately measured. The CAM’s surface was gently lacerated using a sharp debridement spoon in the proximity of a blood vessel bifurcation without causing extensive bleeding. Three methods of implantation were tested, including engrafting the tumor sample with Matrigel and a plastic ring of 5 mm diameter as a barrier (group 1), only with Matrigel (group 2), or native without additional viscoelastic substances or a ring (group 3).

**Figure 1:**
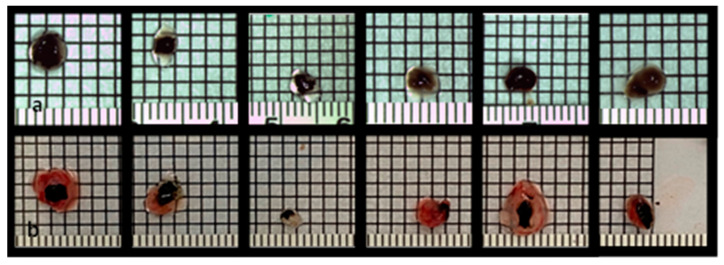
Uveal melanoma patient-derived tumor grafts before implantation onto the CAM on ED7 (**a**) and uveal melanoma nodules on ED18 after dissection (**b**), documentation on a 2 × 2 mm marked sheet.

The plastic rings were created by cutting the tip of inoculating loops (Ino-Loop; Simport Scientific Inc., Saint-Mathieu-de-Beloeil, QC, Canada) and were slightly taped to the periderm on ED7. Thereafter, 25 µL of Matrigel was pipetted either directly onto the previously lacerated area of the CAM or into the middle of the ring. Furthermore, the patient-derived grafts were positioned on ED7 with sharp forceps in the middle of the ring, above the Matrigel droplet, or directly on the CAM accordingly. The tumors were gently pressed and repositioned with the blunt end of the forceps. Moreover, the time interval from enucleation until engraftment was documented. The eggs were then incubated until ED18. The rings were removed on ED8 from the eggs in group 1. The margins of the tumor along the inner circle of the ring were disseminated with forceps and the ring was slowly turned while holding the graft in place with a dental ball burnisher (Figure 1). The growth of the tumor segments was monitored by photodocumentation on ED7, ED8, ED11, ED15, and ED18 with a digital microscope camera (Leica M80, Leica IC80 HD, Leica Biosystems, Nußloch, Germany) and a digital camera (Nikon D60, Nikon, Tokio, Japan), depending on the site of the tumor. On ED18, the embryos were sacrificed by decapitation. The primary implanted tumors, as well as apparent secondary tumors, were excised. The lower CAM was inspected for signs of tumor growth and pigmentation.

### 2.3. Characterization of Tumor Grafts

The tumor grafts were weighed with an analytical balance scale (ABT220-5DM, Kern&Sohn GmbH, Lörrach Germany). The length, width, and thickness of the tumor grafts were measured with a surgical marker ruler on a 2 × 2 mm square laminated sheet. The volume (*V*) and area (*S*) of the fragments were calculated according to the equation for an ellipsoid, where *a*, *b*, and *c* are the lengths of all three semi-axes of the ellipsoid:

*V* = 4/3 π *a b c*.

*S* = *ab*π, *b* ≤ *a*

In order to further assess the formation and growth of the tumors, the circumference was calculated according to the equation for an ellipsoid.

*L* = 4*a*E(*e*), *e* = √1 − (*b*/*a*)^2^, E(*e*): 2nd complete elliptic integral

For the evaluation of changes in shape and, hence, the growth of the tumors, the ellipticity (*c*) and linear eccentricity (*f*) were compared. The ellipticity is a measure of how flattened an ellipse-shaped object is, as well as its deviation from circularity. The eccentricity of an ellipse is less than 1 and indicates how circular the measured object is with reference to a circle. The linear eccentricity (*f*) is the distance between the center and either of two foci. In geometry, foci are special points with reference to which any of a variety of curves is constructed. For the definition of the four types of conic sections, the circle, ellipse, parabola, and hyperbola, two foci can be used to determine the linear eccentricity and the curve type. Therefore, the linear eccentricity adds additional information regarding the shape and growth direction of the examined object.

c = *ba*

*f* = √*a*^2^ − *b*^2^

### 2.4. Measurement Tools

Different measurement instruments were used to generate data regarding the size parameters, vascularization, shape, and distribution of the implanted tumor masses and their alterations during the growth period.

#### 2.4.1. Ultrasound

Ultrasound scans were performed using three ultrasound modalities, an ultrasound biomicroscopy in a water bath with a 40 MHz transducer (VumaxII, Sonomed Escalon, North New Hyde Park, NY, USA), a B-Mode ophthalmic ultrasound with a 10 MHz probe (Ellex, eye cubed, Minneapolis, MN, USA), as well as a B-Mode and color Doppler ultrasound with a 30 MHz 20 mm linear transducer (Vevo 3100, Fujifilm, Visualsonics, Toronto, ON, Canada). On ED18, after removing the Parafilm, the window was widened to ensure access to the tumor grafts. Hylogel (Ursapharm, Saarbrücken, Germany) eye drops or warmed Aquasonic transducer gel (Parker Laboratories, Fairfield, NJ, USA)were placed over the tumor area and on the transducer/probe. Then, the transducer was lowered until contact was established with the gel. Tumors were then visualized along the longitudinal and, if possible, the transversal axes to enable size quantification of the tumors. The image was frozen and the dimensions of the grafts were measured. The Vevo 3100 allowed an additional calculation of the selected area by drawing a line around the tumor’s shape. Examination with the Ellex ophthalmic ultrasound was conducted by placing the probe directly on the tumor or with a fingerstall filled with water above the tumor graft. The fingerstall was created with one finger of a latex glove filled with water, which was knotted and then carefully placed above the tumor. The probe was then slightly pressed against the fingerstall. Furthermore, the color doppler mode enabled the evaluation of the vascularization within the tumor and the assessment of the supplying vessels in the proximity of the tumor.

#### 2.4.2. Optical Coherence Tomography and Infrared Imaging

In vivo tumor confocal infrared (IR) imaging and optical coherence tomography (OCT) were conducted with the spectral domain OCT (Spectralis Heidelberg Engineering, Heidelberg, Germany). The examiner held the egg in a 45 °C degree position without damaging the embryonic structures. The window of the eggshell was expanded if necessary. The infrared images offered an en face perspective and details regarding the vascular supply and provided measurement data for length and width, as well as the marked tumor area. The vertical and horizontal extent, as well as the integration and invasion into the CAM membrane, were evaluated with the OCT images. The largest basal diameter, as well as the depth of invasion, were statistically assessed.

#### 2.4.3. Optical Coherence Tomography Angiography

The OCT–angiography was conducted on ED18 for the imaging and detection of the microvasculature within and at the base of the tumors. The procedure was performed with the Spectralis OCT–angiography module (Heidelberg Engineering) following the steps as described for the SD-OCT. The acquired images allowed the assessment of the supplying vessels of the complete tumor with their surroundings and of the tumor invasion.

#### 2.4.4. Fundus Fluorescein Angiography

For additional information regarding the vasculature of the tumor, 50 µL of Fluorescein was injected intravascularly on ED18 using a 1 mL sterile syringe and a 30-gauge needle. The needle was slightly bent. The syringe was labeled with 50 µL-distance markings and a foldback clip was fixated on the syringe plunger to avoid injecting excess solution. The egg was held at a 45-degree angle to allow the visualization of the tumor and imaging was conducted with the Spectralis Ultra-Widefield fundus angiography module (Heidelberg Engineering).

#### 2.4.5. Image Analysis

The images acquired on ED7 and ED18 were analyzed with ImageJ 1.53 (Dresden, Germany) as a processing program for the multidimensional image analysis. The cross-sectional area, the mean gray value (MGV), and the Feret’s diameter were investigated as a quantitative measurement of mass, density, and distribution to evaluate changes from the first implantation day until the last growth day. In addition, the three-dimensional surface plot plugin for ImageJ was used to generate volumetric images to further enable the visualization of tumor growth as an alternative, non-invasive digital imaging tool.

### 2.5. Histology and Immunohistochemistry

The excised tumor material was transferred into a plastic cassette for immobilization and then placed in buffered formalin (Histofix 4%, Roth, Karlsruhe, Germany) for 24 h. The plastic cassettes were then incubated in PBS before dehydration with a series of graded alcohols and xylene. Each specimen was embedded in paraffin at 58 °C and then cut into 5 µm-thick sections with a rotary microtome (Reichert Jung 2040 Microtome Rotor Slicer, Cambridge Scientific Instruments GmbH, London, UK). The sections were floated in a 56 °C water bath and mounted onto histological slides. The slides were left to dry overnight at room temperature. Subsequently, the slides were rehydrated. Hematoxylin and Eosin (H&E) staining was performed. Furthermore, antigen retrieval was performed using 10 mM EDTA/Tris (pH9) for 30 min in a water bath at 95 °C. Non-specific staining was blocked, incubating in 3% BSA in PBS for 30 min at room temperature. The sections were washed with PBS. The primary antibodies diluted in 1% BSA, 0.3% Triton^®^ X-100, and 0.01% sodium azide in PBS were applied according to the manufacturer’s instructions and incubated overnight at 2–8 °C. In order to identify and mark human uveal melanoma cells, a combination of specific antibodies was applied (combination of anti-HMB45, anti-M2-7C10, and anti-M2-9E3 antibodies; mouse monoclonal; Abcam, Cambridge, UK). Before the application of the secondary antibodies, the slides were washed with PBS for 15 min. Secondary antibodies in combination with the nuclei marker DAPI (4′,6-diamidino-2-phenylindole), diluted in 1% BSA, 0.3% Triton^®^ X-100, and 0.01% sodium azide in PBS, were applied and incubated for 30–60 min at room temperature. Finally, the slides were rinsed with PBS and mounted with anti-fade mounting media. Stained sections were visualized using an Olympus BX51 fluorescence microscope.

### 2.6. Statistical Analysis

The statistical analysis of the data was performed using a two-way ANOVA test and Tukey’s multiple comparisons test (GraphPad Prism 9.4.1 software, GraphPad Software Inc., San Diego, CA, USA). A value of *p* < 0.05 was considered statistically significant and significance levels were indicated as * *p* < 0.05, ** *p* < 0.01, *** *p* < 0.005, **** *p* < 0.001.

### 2.7. Ethics Approval

The ethics committee of the medical faculty of the University Duisburg-Essen approved the study with the number 21-9959-BO. The research was performed in accordance with the Declaration of Helsinki and with relevant local guidelines and regulations.

## 3. Results

### 3.1. Characterization of Tumor Grafts

**Figure 2:**
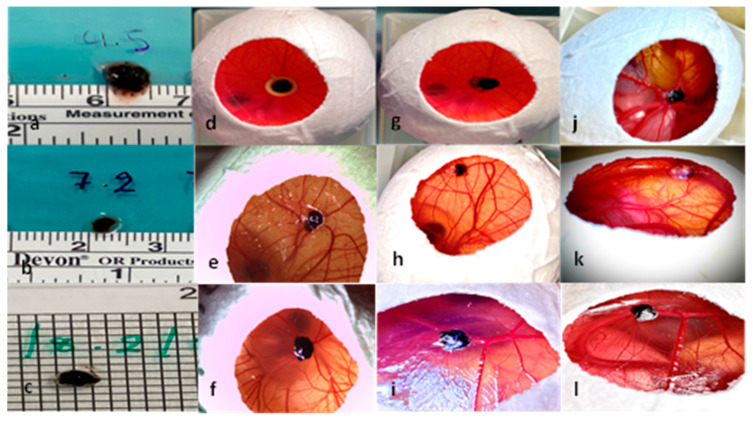
Presentation of the uveal melanoma patient-derived tumor graft development in the three experimental groups. (**a**–**c**) Uveal melanoma patient-derived grafts before implantation, (**d**) implantation of tumor sample with Matrigel and a ring on ED7, (**e**) implantation of a tumor sample with Matrigel without a ring on ED7, (**f**) implantation of a tumor graft without Matrigel or a ring on ED7, (**g**) tumor graft on ED8 after ring removal with loss of cohesion and dissociation of the tumor margins, (**h**) tumor graft on ED8 after implantation only with Matrigel, (**i**) tumor graft on ED8 after implantation without Matrigel or a ring, (**j**) tumor growth on ED18 in group 1 with Matrigel and a ring, (**k**) tumor growth on ED18 in group 2 with Matrigel, and (**l**) tumor growth on ED18 in group 3 without Matrigel or a ring.

A total of 46 eggs were implanted with uveal melanoma patient-derived grafts. The implantation of the tumor fragments was conducted with Matrigel and a plastic ring in 15 eggs (group 1), with Matrigel without a ring in 16 eggs (group 2), and natively without Matrigel nor a ring in 15 eggs (group 3) (Figure 2). The mortality varied among the three groups, with 20%, 18.75%, and 20%, respectively, in groups 1, 2, and 3. One egg in group 3 was accidentally damaged during the experimental procedure. There were no significant differences among the groups (20%, 25%, and 26.6%) regarding the transposition of the grafts from their original implantation site, while complete descendance into the lower CAM only occurred in group 1.

**Figure 3:**
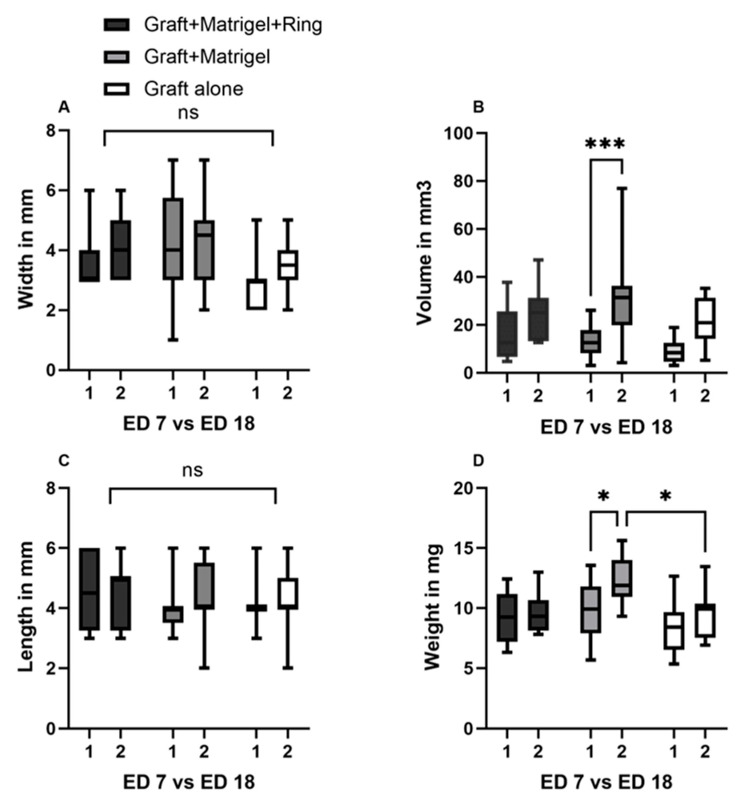
Characterization of the implanted uveal melanoma patient-derived grafts: comparison of length, width, volume, and weight between ED7 and ED18, as well as among groups 1, 2, and 3. (**A**) Width on ED7 and ED18 in groups 1, 2, and 3, (group 1: graft + Matrigel + ring, group 2: graft + Matrigel, and group 3: graft alone). No significant differences could be seen between the tested conditions or during the experimental period. (**B**) Calculation of volume on ED7 and ED18 in the three tested groups. A significant increase in volume was documented in the group implanted with Matrigel alone. (**C**) Length of the tumor grafts on ED7 as well as on ED18 with no significant differences between the groups and during the growth period. (**D**) A statistically significant difference in weight was only measured in group 2 between ED7 and ED18. In addition, the weight difference on ED18 after excision between groups 2 and 3 was statistically significant. Statistical analysis was performed using a two-way ANOVA and Tukey’s multiple comparisons test. Significance levels are indicated with * *p* < 0.05, *** *p* < 0.001, ns = non significant.

In group 1 the implanted grafts showed a mean length of 4.6 mm (±1.3 SD), a mean width of 3.7 mm (±1.1 SD), and a mean thickness of 1.4 mm (±0.4 SD) before implantation, and 4.5 (±1.08 SD), 4.1 mm (±1.06 SD), and 2.5 mm (±0.5 SD) after dissection. The same parameters had values of 4 mm (±0.8 SD), 4 mm (±1.7 SD), and 1.5 mm (±0.4 SD) before implantation and 4.3 mm (±1.1 SD), 4.2 mm (±1.3 SD), and 2.9 mm (±0.6 SD) on ED18 for group 2. In the group without Matrigel or a ring, a mean length, width, and thickness of 4.1 mm (±0.75 SD), 2.8 mm (±0.9 SD), and 1.4 mm (±0.4 SD) were measured on ED7 and 4.3 mm (±1.2), 3.5 mm (±0.8 SD), and 2.5 (±0.3 SD) on ED18, respectively. In all groups, the length and width showed an increase between ED7 and ED18, which was not significant (Figure 3A,C). A statistically significant difference (*p* = 0.0007) between the volume on ED7 and ED18 could only be seen in group 2 with Matrigel (Figure 3B). Furthermore, a statistically significant increase in weight was documented in the same group during the development process (*p* = 0.0216). Among the tested groups, there were no significant differences in length, width, or volume, whereas the weight on ED18 varied significantly between groups 2 and 3 (*p* = 0.0218) (Figure 3D).

**Figure 4:**
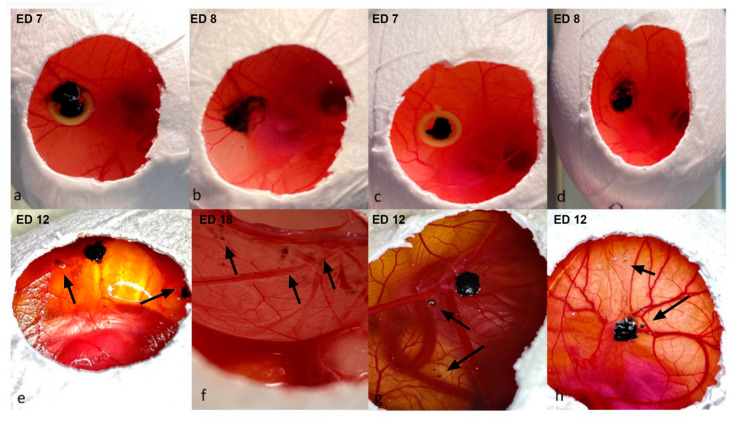
Detachment of the peripheral sections of the implanted uveal melanoma grafts, (**a**–**d**): implantation with Matrigel and a ring (group 2), (**e**–**h**) implantation without Matrigel or a ring (group 3). (**a**,**c**) Images of the implanted uveal melanoma patient-derived grafts with Matrigel and a ring on ED7 directly after engraftment; the larger tumor sample does not fit inside the ring, as depicted in images a, b, and d—dissociation and loss of cohesion in the periphery of the tumors directly after the removal of the rings on ED8. (**e**,**g**,**h**) Loss of cohesion and detachment of tumor segments from the main tumor body on ED12 in group 2, as indicated with arrows. (**e**,**f**) Invasion of the tumor depicted in image e into the lower CAM with loss of peripheral cohesion after the dissection of the embryo on ED18.

The tumors implanted with Matrigel and ring (Figure 4a,c) showed a higher detachment of the peripheral sections of the grafts after the removal of the rings on ED8 (Figure 4b,d), whereas a similar loss of cohesion, resulting in the detachment and migration of fragments apart from the main tumor body, was noted in group 3 after ED12 (Figure 4e,g,h). Pigmentation of the lower CAM was predominantly documented in group 3 (0%, 12.5%, and 33.3%) (Figure 4f).

**Figure 5:**
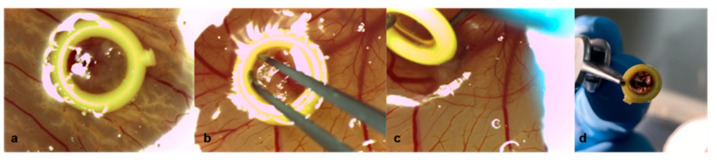
Removal of the rings on ED8 of uveal melanoma patient-derived tumor grafts in group 1 with Matrigel and a ring. (**a**) Tumor graft with Matrigel and a ring on ED7, (**b**) dissemination of the tumor margins along the inner circle of the plastic ring with forceps on ED8, (**c**) slowly turning and lifting the ring with a forceps while holding the graft in its implantation site with a ball burnisher to avoid involuntary explantation on ED8, (**d**) removed ring with fragments of the tumor on ED8.

The extended perseverance of the rings until removal was associated with a higher dissociation of the tumors. In group 1, fragments of the tumor mass remained adherent to the inner circle of the rings while lifting the rings, despite careful surgical dissemination of the margins (Figure 5a–c). Furthermore, larger tumor samples did not fit and could not be adjusted inside the ring, resulting in damage to parts of the implanted grafts during ring removal (Figure 4a,b).

**Figure 6:**
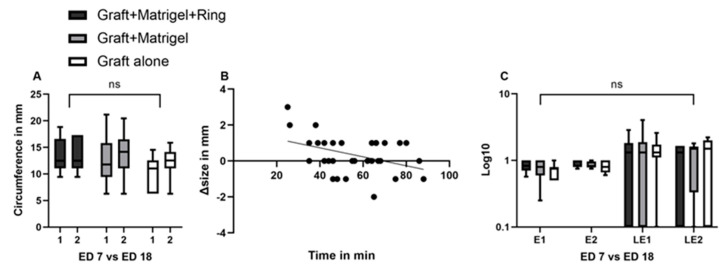
Characterization of uveal melanoma patient-derived grafts: comparison of circumference, ellipticity, and linear eccentricity between ED7 and ED18, as well as among groups 1, 2, and 3. Association and effect of time interval on tumor size. (**A**) A non-significant increase was documented among the three groups or during the growth period for each group, (**B**) linear regression of the time interval between enucleation and engraftment onto the CAM and the difference in size between ED7 and ED18, (**C**) a non-significant increase was documented regarding the ellipticity and the linear eccentricity of the tumor grafts during the growth period and among the tested groups. A Log10 scale was used for the Y axis. Statistical analysis was performed using a two-way ANOVA and Tukey’s multiple comparisons test. ns = non significant.

In addition, an association of the size difference before implantation and after excision was noted with the time interval between enucleation and engrafting onto the CAM (Figure 6B). A longer interval until implantation led to either the dissolution of the grafts when preserved in medium in a tube or to the decomposition of the grafts when maintained in a petri dish with a small amount or without any medium, both resulting in the loss of tumor fragments during positioning onto the CAM.

A non-significant increase in circumference was documented between ED7 and ED18 in groups 2 and 3 (Figure 6A). The mean circumferences were 12.4 mm (±4.2 SD) and 10.2 (±3 SD) on ED7 and 13.5 (±3.8 SD) and 12.3 (±2.9 SD) on ED18 for groups 2 (*p* = 0.96) and 3, respectively (*p* = 0.63). Less prominent changes were noted in ellipticity and linear eccentricity among all groups and during the growth period, resulting in statistically non-significant differences (*p* > 0.99) (Figure 6C).

### 3.2. Measurement Tools

#### 3.2.1. Ultrasound

**Figure 7:**
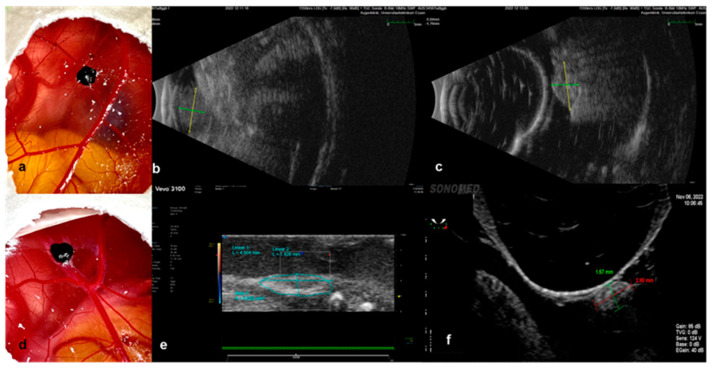
Ultrasound as a monitoring instrument to evaluate the growth of the implanted tumors in the CAM Model. (**a**–**c**) Examples of the evaluation of size with the B-Scan ophthalmic ultrasound, where the tumor, implanted with Matrigel, was measured directly with the 10 MHz probe; it presents as a hyperechoic ellipsoid mass on the surface of the CAM, (**b**) and subsequently with the latex fingerstall filled with water for the adjustment of depth as well as for the acquisition of images of higher definition. (**c**–**f**) Examples of the evaluation of graft size via a preclinical imaging system with ultra-high-frequency ultrasound (**e**) and ultrasound biomicroscopy (**f**) after implantation without Matrigel.

The largest basal diameter as well as the cross-sectional area were calculated via sonographic evaluation in 25 eggs. The conduction of an ultrasound was possible in 25 eggs, depending on the site of the tumor. Grafts that migrated in the periphery or along the shell on the CAM were not accessible and were excluded from sonographic measurements. The implanted grafts in the CAM as well as their measurements with the tested ultrasound modalities are presented in Figure 7a–f. The cross-sectional area of the examined implants was only estimated with Vevo3100 (Figure 7e). Another advantage of the ultra-high-frequency ultrasound was the evaluation of the vessels inside the tumor, as well as the efferent and afferent vessels in the proximity of and along the tumor base. The signal was more intense along the margins for all tested eggs, whereas a strong signal with multiple visible vessels centrally was detected in 76% of the examined grafts. Due to the size of the transducer of the Vevo 3100 ultrasound, a transversal evaluation was feasible in a restricted number of eggs due to space limitation and potential damage to the eggshell. The B-Scan ultrasound biomicroscopy in a water bath was associated with a higher risk of injury to the embryos and the grafts due to the movement of the tip, which further impeded the stability of the examination conditions (Figure 7f). The B-Scan with the device for ophthalmic ultrasound was conducted with the 10 MHz probe directly on the tumor, as well as with the use of a fingerstall filled with water to adjust the depth and utilize the posterior acoustic enhancement to achieve more defined images (Figure 7b,c). In all sonographic modules, the tumor samples presented as hyperechoic ellipsoid masses on the surface of the CAM. The homogeneity of the echo’s intensity differed depending on the size of the grafts, while tumors implanted with Matrigel displayed a more homogenous profile. The size parameters did not show a significant difference among the various tested ultrasound modalities.

#### 3.2.2. Infrared Imaging, Optical Coherence Tomography and Angiography, and Fluorescein Angiography

**Figure 8:**
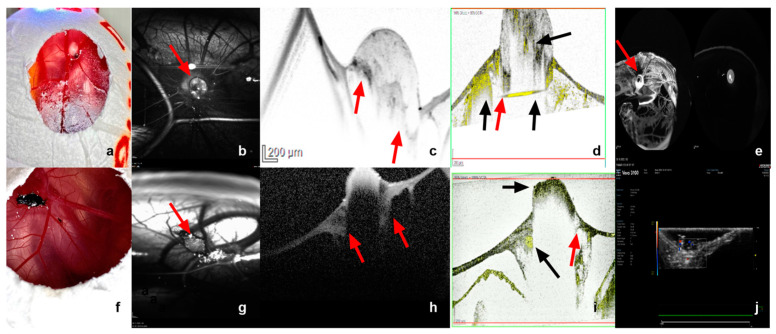
Infrared imaging, OCT and OCT–angiography, fluorescein angiography, as well as color doppler sonography as monitoring tools for tumor growth and vascularization. (**a**–**e**) Tumor graft implanted with Matrigel alone on ED18, (**f**–**j**) tumor graft implanted without Matrigel on ED18. (**b**,**g**) Visualization of the tumor (arrow) as well as the detailed vascular supply in the proximity and around the base of the tumor via infrared imaging, presentation of the typical vascular star around the implanted graft. (**c**,**h**) Monitoring of the tumor size and invasion of the tumor into and under the CAM membrane as a sign of growth and successful implantation with optical coherence tomography. (**d**,**i**) Optical coherence angiography for the evaluation of the microvasculature inside the tumor and, therefore, of the viability, visualization of an intense vascular signal around the graft and under its base, as already shown in the infrared imaging, and invasion and growth of the tumor into the CAM; red arrow indicates the sign of invasion into the CAM and black arrows indicate vascular supply in the tumor tip, base, and around the graft. (**e**) A precise visualization of the vascular plexus of the complete CAM, as well as around the tumor (arrow) after intravascular injection of 50 µg fluorescein in a supplying vessel of the tumor. (**j**) Color doppler ultrasound as a further alternative non-invasive instrument to control the vascularization inside the grafts and their viability.

The size and shape characteristics of the implanted patient-derived grafts (Figure 8a,f) were additionally monitored and visualized with optical coherence tomography and infrared imaging. The optical coherence tomography and the angiography enabled the measurement of the largest basal diameter (LBD), as well as the thickness of the growing uveal melanoma tumor grafts (Figure 8c,h). Furthermore, the invasion of the tumor samples into the CAM was precisely depicted and measured in both OCT and OCT–angiography (Figure 8c,d,h,i). Besides the color doppler ultrasound, optical coherence angiography and fluorescein angiography were conducted for the evaluation of the vascularization and, therefore, the viability of the implanted uveal melanoma grafts (Figure 8d,e,i,j). Infrared images could be acquired for all viable embryos and tumors, offering not only information on the size and area, but also regarding the blood supply of the grafts, accurately depicting the vascular star around the tumors and the intensified ring-shaped signal at their base (Figure 8b,g). The OCT and angiography were carried out on 22 eggs, depending on the tumor site. The central localization of the tumors was considered an optimal site for the monitoring of the engraftment. Nevertheless, a more marginal position enabled easier access and an unhindered scanning process for the OCT and the angiography. The tilting of the eggs in front of the OCT device led to fold formation in the CAM, which often hid the tumors, especially when localized centrally, and did not allow their imaging. Intratumoral and marginal vessels were detected in all tested eggs. The optical coherence angiography exhibited a more intense signal for neovascularization along the base of the tumors that were implanted with Matrigel (Figure 8d). Such an intense signal could be clearly visualized in 42%, 75%, and 57% of the tested grafts in groups 1, 2, and 3, respectively. Whereas these non-invasive monitoring techniques provide us with sufficient data with regard to the graft blood supply, the complete plexus of the CAM with the vascular star in the proximity of the tumor could only be evaluated and visualized after the intravascular injection of fluorescein (Figure 8e).

**Figure 9:**
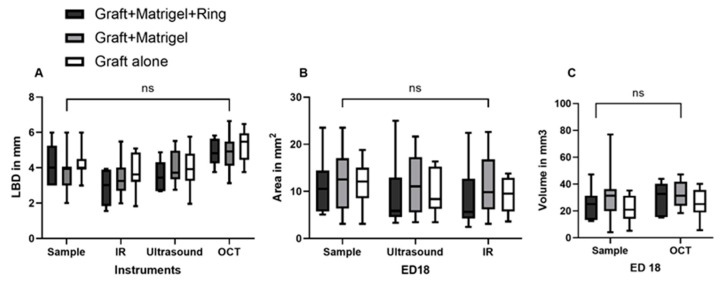
Comparison of the largest basal diameter, the area, and the volume of the implanted uveal melanoma patient-derived grafts, measured with various tested monitoring instruments. (**A**) The largest basal diameter (LBD) of the tumor grafts in all three tested groups of implantation (group 1: graft + Matrigel + ring, group 2: graft + Matrigel, and group 3: graft alone) as calculated on ED18 for the excised samples with infrared imaging (IR), ultrasound, and optical coherence tomography (OCT). (**B**) The cross-sectional area of the tumors was measured by ultrasound and IR. (**C**) The volume calculated with the size parameters of the excised samples, as well as with the OCT measurements, regarding depth invasion. Statistical analysis was performed using a two-way ANOVA and Tukey’s multiple comparisons test. ns = non significant.

The mean LBD values were calculated to be 2.8 mm (±0.9 SD), 3.4 mm (±1.1 SD), and 3.8 mm (±1 SD) with IR, 3.5 mm (±0.9 SD), 4 mm (±1 SD), and 3.9 mm (±1.1 SD) with ultrasound, and 4.9 (±0.8 SD), 4.8 mm (±1 SD), and 5.2 mm (±0.9 SD) with OCT on ED18 for groups 1, 2, and 3, respectively. No statistically significant differences could be detected with respect to the LBD among the measurements of the samples after dissection and the various tested monitoring modalities (Figure 9A). The cross-sectional area was calculated mathematically based on the size parameters of the excised fragments and further compared with the calculation of area via ultrasound with the Vevo3100 device, as well as with infrared imaging. Significant differences were not detected among the tested monitoring modalities regarding the cross-sectional area (Figure 9B). Moreover, the volume was calculated based on the excised samples’ size parameters and was compared with the volume measured with data from the OCT assessment. No significant differences could be documented among the tested groups and instruments (Figure 9C).

#### 3.2.3. Image Analysis Using Image J

**Figure 10:**
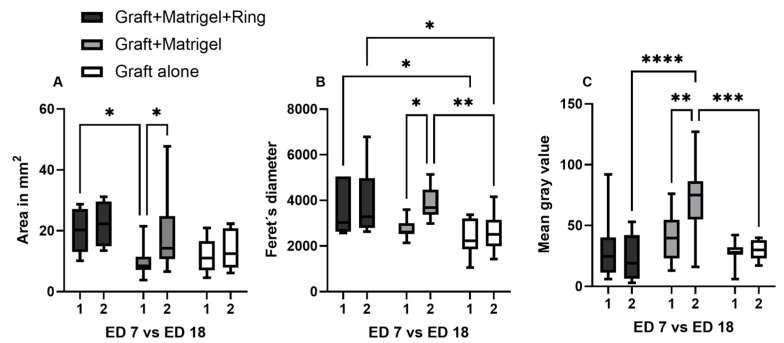
The cross-sectional area, Feret´s diameter, and mean gray value of the implanted uveal melanoma patient-derived tumors on ED7 and ED18 via Image J. (**A**) Comparison of the calculated cross-sectional area of the grafts between the three tested implantation techniques (group 1: graft + Matrigel + ring, group 2: graft + Matrigel, group 3: graft alone) and during growth period from ED7 to ED18 in each group, (**B**) calculation of the Feret´s diameter as a mass distribution parameter and comparison among the three tested groups and during the growth period from ED7 to ED18 in each group, (**C**) the mean gray value was measured as an indicator of tumor density among the three tested groups and during the growth period from ED7 to ED18 in each group. Statistical analysis was performed using a two-way ANOVA and Tukey’s multiple comparisons test. Significance levels are indicated with * *p* < 0.05, ** *p* < 0.01, *** *p* < 0.001, and **** *p* < 0.0001.

Besides the comparison of the calculated cross-sectional area on ED18 in comparison with other monitoring tools, such as ultrasound and infrared imaging, the values on ED7 and ED18 were analyzed using Image J to further evaluate changes during the growth period using digital image processing. Significant differences were detected with reference to the calculated cross-sectional area via Image J on ED7 between the group with Matrigel and a ring and the group with Matrigel alone (*p* = 0.02). A significant increase in the measured area during tumor growth between ED7 and ED18 was only observed in the second group with Matrigel alone (*p* = 0.04) (Figure 10A).

The Feret’s diameter is a measure of an object’s size along a specified direction. It was measured with Image J, as an additional size parameter, to quantitatively compare the three tested implantation techniques and to further investigate the dimensional changes and mass distribution during the tumor attachment and growth into the CAM. A significant deviation of values was demonstrated for group 3 compared with group 1 on ED7 (*p* = 0.03) and compared with both groups 1 (*p* = 0.01) and 2 (*p* = 0.004) on ED18. Between ED7 and ED18, a significant difference was estimated only for group 2, displaying an apparent increase in size for the grafts implanted with Matrigel alone (*p* = 0.01) (Figure 10B).

The mean gray value is indicative of the tumor density; here, it was calculated based on photographic imaging on ED7 and ED18. The generated data for group 2 on ED18 exhibited higher values with a significant difference, compared with groups 1 (*p* < 0.0001) and 3 (*p* = 0.0001). Moreover, the mean gray value significantly increased from ED7 to ED18 in group 2 (*p* = 0.005) (Figure 10C).

**Figure 11:**
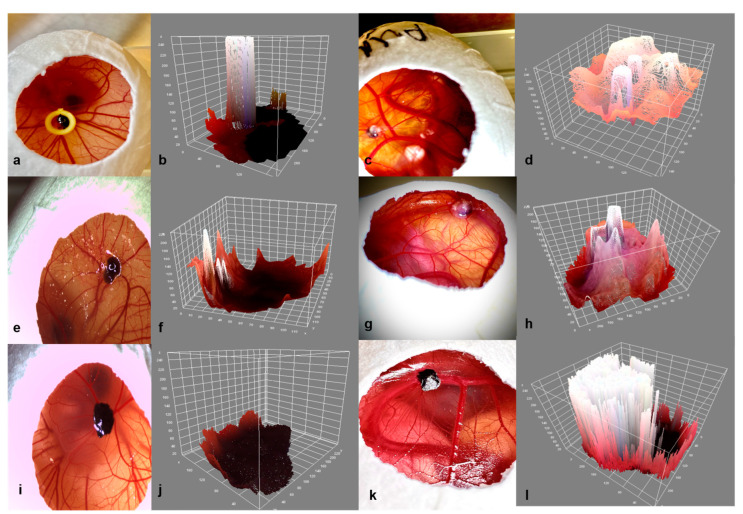
Three-dimensional visualization of the tumors on ED7 and ED18 in the three experimental groups. (**a**–**d**) Graft + Matrigel + ring, (**e**–**h**) graft + Matrigel, (**i**–**l**) graft alone, (**b**,**f**,**j**) 3-D reconstruction of the implanted tumor grafts on ED7 for groups 1, 2, and 3, (**d**,**h**,**l**) 3-D reconstruction of the implanted tumor grafts on ED18 for groups 1, 2, and 3 using the three-dimensional surface plot plug-in in Image J.

The three-dimensional surface plot plugin was investigated as an alternative non-invasive instrument for the evaluation of mass expansion and volume increase during the development phase and for the detection of their differences among the three tested groups (Figure 11a–l). The uveal melanoma grafts implanted into the CAM with Matrigel with or without a ring displayed a greater increase in volume in comparison with group 3 without viscoelastic support (Figure 11d,h,l). On the other hand, tumors in group 1 with Matrigel and a ring showed a lower mass density in comparison with group 2 with Matrigel alone (Figure 11d,h). Furthermore, all groups formed ellipsoid tumors, while the grafts implanted with Matrigel developed an almost conic arrangement of the tumor tip and exhibited a more inhomogeneous configuration.

#### 3.2.4. Histology and Immunohistochemistry

**Figure 12:**
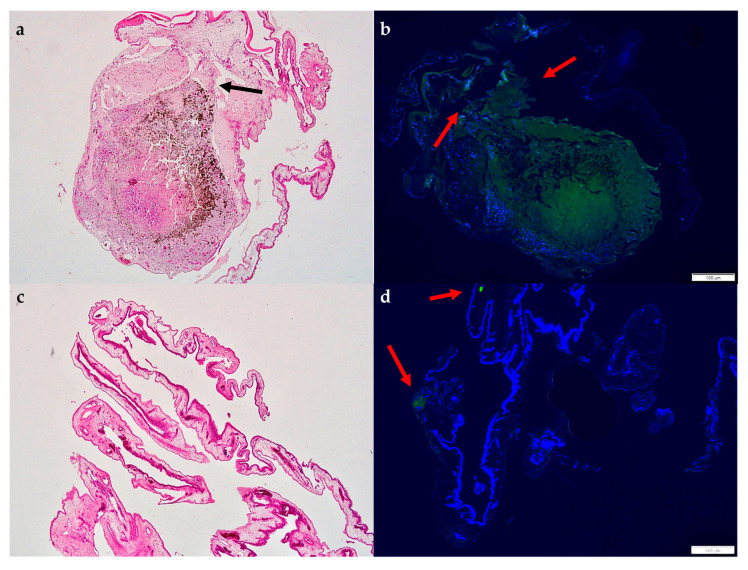
Images of H&E staining and fluorescence microscopy of an excised patient-derived UM-xenograft, as well as of the lower CAM. (**a**) Hematoxylin and Eosin staining of the tumor nodule implanted with Matrigel with a depiction of the folding of the CAM around the tumor graft, as well as the dispersion and invasion of the pigmented cells in depth under the tumor base (arrow), (**b**) fluorescent microscopy after the application of melanoma-specific staining (green) for the identification of uveal melanoma cells (arrows), scale 100 µm, (**c**) hematoxylin and eosin staining of the lower CAM, (**d**) fluorescent microscopy of the lower CAM with weakly visible signaling of melanoma cells (arrow), scale 100 µm.

The assessment of the histologic specimens after H&E staining (Figure 12a,c) resulted in significantly smaller tumor size parameters compared with the samples and the other applied monitoring instruments. When the LBD was compared with the excised sample data, a significant reduction of approximately 30% was documented in the tested implantation groups. A significant difference in the size parameters was not detected histologically among the used grafting techniques. However, the distribution of the cells in depth, as well as the encasing of the tumor within the CAM as signs of integration and invasion, could be clearly visualized in H&E images, as well in the fluorescence microscopy (Figure 12b,d).

## 4. Discussion

The development of in vivo models that can precisely capture the heterogeneity of cancer characteristics and their response to new therapeutic agents is a crucial prerequisite for proficient translational oncology and improvement in patients’ prognosis. Existing animal models for uveal melanoma exhibit limitations, each representing available in vivo options for a specific scientific question. The delayed research progress may be attributed to these limitations and the lack of an integral animal model for uveal melanoma, leading to a dismal improvement in prognosis, despite the advances in and the refinement of the treatment procedures of the primary tumors [41]. PDX platforms facilitate the investigation of genetic data and the prediction of the sensitivity of an individual patient’s cancer to specific treatments due to the retainment of the pathophysiology and molecular characteristics of the donor tumor. Vascular changes and invasion are critical factors in most cancer entities, including uveal melanoma, as the tumor’s growth and extension may influence the selection of a surgical treatment and, therefore, severely affect the quality of life for patients with ocular melanoma. The avian CAM constitutes a feasible model for the inoculation of tumor cells as, well as for the implantation of patient-derived grafts, offering valuable information regarding the evaluation of target pathways and treatment response in a cost- and time-effective manner. The current study describes a novel method for establishing and monitoring patient-derived xenografts in fertilized eggs by implanting uveal melanoma patient tumor fragments on the CAM.

PDX models bearing implanted tumors from human tissue, as replicates for the diversity of human malignancies, are increasingly being investigated to meet the demands of precision medicine. Various uveal melanoma models have been developed, mainly via the inoculation of established human cell lines obtained from primary or metastatic tumors into animals, more commonly in mice, rats, rabbits, and CAM [38,42,43]. Nevertheless, there are limited reports of successful PDX in vivo models for uveal melanoma. Nemati et al. transplanted fresh tumor samples obtained after enucleation into the interscapular fat pad of two to four non-preirradiated immunodeficient mice without any extracellular matrix preparation. They postulated that the origin of the tumor was the only factor that was significantly correlated with tumor take [39]. Another group attempted the establishment of a serially transplantable PDX uveal melanoma model in mice and considered the stroma content of the tumors as an influencing factor for the take rate [44]. In the study by Kageyama, an orthotopic PDX mouse model from uveal melanoma hepatic metastasis was developed, which was subsequently monitored via CT imaging before resection and histological evaluation [45].

The chick embryo model has been used only in a limited number of studies for uveal melanoma to date, which do not address the implantation of patient-derived samples. Despite the short period for tumor growth, the CAM can serve as a reliable model for the investigation of multiple components of uveal melanoma biology. Kalirai et al. successfully established an UM model following UM92.1 cell grafting onto the CAM, assessing the ability of uveal melanoma cells to undergo orthotopic growth in the chick eye, form tumor masses on the CAM, and undergo dissemination via the chick’s circulation to internal organs [38]. An optimized protocol was described for the inoculation of UM 92.1, UPMD2, UPMM3, and Mel270 cell lines onto the CAM in order to increase the viability of the embryos and the implanted grafts [43]. Moreover, the antitumoral effect of electrochemotherapy on uveal melanoma was investigated in the CAM after the implantation and sufficient growth of tumor organoids from UM92.1 cell lines [46]. The CAM assay is an easily accessible model that facilitates the direct observation and precise monitoring of tumor growth, as well as the rapid assessment of the effectiveness of novel candidate therapeutic drugs. In addition, the direct microscopy of the chorioallantoic microcirculation may reveal signs of metastasis formation and intravasation. A study described the identification of labeled melanoma cells via intravital videomicroscopy and epifluorescence to study in vivo metastasis formation [40]. The CAM itself is not innervated and experiments are terminated before the development of centers in the brain associated with pain perception; therefore, CAM studies are not considered animal experimentation and do not require approval by an animal welfare or ethics committee in Germany and in most European countries [47]. However, the short observation period impedes the production of macroscopically visible metastasis in chick tissues. In our study, the growth and extension of pigmented cells to the lower CAM could be evidently visualized on ED18 in a limited number of eggs. A prolongation of the experimental period could be achieved by re-grafting implanted cells or tissue onto the CAM of another egg for the assessment of tumor extension and long-term treatment responses. Nonetheless, studies suggest that tumors on the CAM show more rapid development than equivalent subcutaneous grafts in mice [48,49]. Further disadvantages of the chicken embryo are the high mortality rate and the differences observed in the embryonic membranes, as well as in the composition of the amniotic fluid. Another limitation of this in vivo model is the occurrence of non-specific inflammatory reactions, despite the natural immunodeficiency of the chick embryo, if experiments are extended after 15 days of incubation. In spite of the potential disadvantages, the CAM assay is a versatile model, offering a translational significance analogous to that of rodent experiments for specific oncological research questions.

In the present study, the invasion of the ectoderm, the migration to the mesoderm, and merely the penetration of the endoderm, as well as the highly vascularized surrounding tumor area, could be documented intravitally through OCT and OCT–angiography. The invasion of PDX resulting in the destruction of the ectoderm, the subsequent migration into deeper avian tissue structures, and the metastatic dissemination through intravasation has already been described in various cancer entities in PDX CAM models, such as in hepatocellular carcinoma, laryngeal cell carcinoma, urological cancer, and renal cell carcinoma [50,51,52]. Tumor graft growth and size characterization constitute a critical step of validation and successful engraftment. Hu et al. showed that the CAM xenograft maintains the same tumor growth pattern and metastatic behavior as that observed in mice [51]. In most PDX experiments, three-dimensional size monitoring and, therefore, the calculation of the thickness or depth of invasion is achieved using calipers or advanced imaging, such as CT, MRI, or bioluminescence detection [37,53,54,55]. In various studies, the tumors‘ dimensions and size characteristics were estimated by utilizing an endpoint size or weight measurement [48,56]. Histological assessment is considered as the gold standard; however, inaccuracies can arise due to deviations in the sectional plane, artifacts, folding, and shrinkage during the preparation of histological sections [37]. Eckrich et al. reported that the size measured histologically was much lower than that evaluated sonographically due to the intense shrinkage of the specimen during the fixation process. Photographic documentation after excision was used to further compare and validate the ultrasound size measurements, showing similar data, exceeding the measurements obtained from histological specimens. The viability of the eggs incubated with tumors, as well as sufficient ingrowth of the transplanted grafts, was approximately 80% in our study. In the literature, a wide range of take rates from 40 up to 80% has been reported, depending on the tumor entity [55,57,58]. The discrepancy in the reported rates can be attributed to the death of the chicken embryo, luxation, transposition of the graft to the eggshell, and signs of insufficient ingrowth or vascularization.

Alternative imaging techniques, such as MRI or CT, are also applicable for the assessment of tumors on the CAM. The evaluation of soft tissues with CT would require the administration of contrast agents for the estimation of tumor growth. On the other hand, high-resolution imaging via MRI is associated with practical limitations, such as the significant increase in time and cost factors, as well as impeded acquisition of accurate measurements due to the movement of the embryo. Zou et al. proposed an age-adapted cooling regime for the immobilization of the chick embryo, enabling high-resolution MRI of 15 embryos after the xenotransplantation of human MDA-MB-231 breast cancer cells on the CAM [59]. They also investigated the applicability of in ovo MRI for the monitoring of MR contrast agent-labeled compounds in mamaria carcinomas xenotransplanted onto the CAM after the systemic injection of the compounds into a chorioallantoic capillary vein, assessing their biodistribution in different organs of the chicken embryo, as well as in xenotransplanted tumors at all time points [60]. Another group quantified subtle differences in morphogenesis, acquiring highly detailed, quantitative 3D datasets of embryonic chicks with Micro-CT [61]. In ovo imaging for the longitudinal direct quantification of the biodistribution of compounds or monitoring of surrogate markers via MRI and PET provides detailed anatomical and functional information, as well as excellent sensitivity accordingly [62].

Significant differences in size parameters, such as length and width, could not be detected between the three different implantation techniques in the present study. The slow growth rate of uveal melanoma must be taken into consideration for the interpretation of the non-significant increase in the size parameters within all three groups [63]. Nevertheless, a significant increase in volume and weight was only identified in the group of grafts implanted with Matrigel alone during the development period. These findings, in combination with the apparent dissociation of the uveal melanoma tumors during the ring removal in group 1 or the loss of cohesion in group 3, underline the advantages of engraftment only with Matrigel. Furthermore, image analysis with Image J, based on the photographic documentation, also supports the hypothesis of more apparent growth between ED7 and ED18 within group 2. However, the image analysis showed differences between the groups that were not detected with other monitoring instruments and could be attributed to the documented dissociation of the tumors and, therefore, a false increased calculation of the tumor area in groups 1 and 2. The three-dimensional analysis of the tumor surface, as shown in Figure 11, constitutes a simple supplementary tool for the rapid assessment of the structural behavior of the grafts, based only on photodocumentation. Infrared imaging is a further non-invasive option for the generation of measurement data regarding growth and a detailed presentation of vascular expansion. The correlation of the time interval, between enucleation and implantation, with the difference in size throughout the growth phase emphasizes potential alterations of the specimen’s consistency with time and the significance of a reciprocal coordination of both the surgical and study teams for rapid implantation.

All applied real-time imaging techniques delivered comparable results with no significant differences for the evaluation of various growth parameters, potentially offering the prospect of repetitive measurements due to their non-invasive nature. The different tested ultrasound modalities were accompanied by distinctive advantages and disadvantages, while the frequency of damage to the embryo or the underlying tumor graft was higher with ultrasound biomicroscopy. A more precise approach for the assessment of tumor growth and invasion was ensured through the conduction of an OCT. The limited accessibility of central xenografts was attributable to the necessary tilting of the egg during the scanning process and the fold formation of the CAM surface. Furthermore, this study investigates multiple methods for the assessment of vascularization. The advantages of the examination of human tumor fragments on CAM and their perfusion using color doppler and a rating system for intratumoral vascularization have already been described [37]. Optical coherence angiography offers high-resolution imaging and accurately incorporates multidimensional monitoring and a precise evaluation of invasion and vascular expansion, not only intratumorally, but also in the proximity of the tumor, indicating its precedence in comparison with color doppler ultrasound. The injection of fluorescein is an invasive technique for the direct visualization of the tumor vascular supply, as well as the depiction of the complete plexus of the CAM. It cannot identify intratumoral microvessels with the precision and imaging quality of optical coherence angiography. Moreover, the embryos must be immediately sacrificed after the injection of fluorescein, prohibiting the use of these techniques for the repetitive evaluation of perfusion.

## 5. Conclusions

We demonstrated the successful establishment of a novel uveal melanoma patient-derived xenograft model based on the CAM assay, analyzing different implantation and monitoring techniques. Despite its limitations, especially the short observation period, the chick CAM assay represents a valuable cost- and time-efficient in vivo system, that does not require the approval of an ethics committee and can potentially be used as an intermediate step for the preliminary testing of new drug agents prior to animal experimentation. Incongruences in tumor environments and immune properties are minimized with PDX models, while the high efficiency of PDX engraftment on CAM and the increasing interest in the research community highlight the necessity for precise in vivo monitoring instruments for the evaluation of tumor growth and vascularization in the CAM. Moreover, real-time imaging with multiple comparable modalities expands the applicability of the CAM assay as a PDX model in experimental oncology.

## Data Availability

The data presented in this study are available in this article.
